# Smear Layer and Debris Removal from Root Canals Comparing Traditional Syringe Irrigation and 3D Cleaning: An Ex Vivo Study

**DOI:** 10.3390/jcm12020492

**Published:** 2023-01-06

**Authors:** Alfredo Iandolo, Massimo Pisano, Dina Abdellatif, Giuseppe Sangiovanni, Giuseppe Pantaleo, Stefano Martina, Alessandra Amato

**Affiliations:** 1 Department of Medicine and Surgery, University of Salerno, 84081 Salerno, Italy; 2 Department of Endodontics, Faculty of Dentistry, University of Alexandria, Alexandria 21531, Egypt

**Keywords:** EDTA, endodontics, root canal irrigants, smear layer, sodium hypochlorite, ultrasonics

## Abstract

Background: Endodontic treatment objectives comprise eliminating or decreasing bacterial load inside the complex endodontic space. Removing the smear layer and debris becomes mandatory to achieve good three-dimensional (3D) cleaning. Aim: This study assesses the difference in smear layer removal using the 3D cleaning technique and traditional syringe needle irrigation. The 3D cleaning technique includes the ultrasonic activation of intracanal-heated NaOCl. Materials and Methods: Our current study used single-rooted human mandibular premolar teeth to test the earlier-mentioned technique (*n* = 30). Initially, an endodontic access cavity was performed. Consequently, specimens were randomly distributed into three study groups according to irrigation protocol. The groups were Group 1, where the traditional syringe needle irrigation system was applied; Group 2, where the 3D cleaning technique was performed; and Group 3, in which teeth remained uncleaned as it was regarded as the control group. Once the experiment was completed, the teeth were decoronated at the cementoenamel junction (CEJ) and examined using scanning electron microscopy (SEM). Debris and smear layers were viewed in 1000× magnification and scored. Results: Statistical analysis was performed with a standard statistical software package (SPSS, version 28.0; SPSS IBM, Armonk, NY, USA). Data were analyzed with a nonparametric analysis of variance (Kruskal–Wallis ANOVA) among the groups tested and among the thirds of the canals. The level of significance was set at *p* < 0.05. A statistically significant (*p* < 0.05) lower mean smear layer and debris score was observed in both study groups compared to the control group. Group 2 showed better results compared to Group 1. Conclusions: The present study concluded that the 3D cleaning technique is an effective irrigation method for removing debris and smear layers. Future research, such as CLSM (Confocal Laser Scanning Microscopy) and Histological study, should be employed to confirm this study’s conclusion.

## 1. Introduction

This study intends to evaluate the difference in smear layer and debris removal using the 3D cleaning technique and traditional syringe needle irrigation.

Chemical cleaning is considered an important phase in root canal treatment; in this stage, the irrigants should thoroughly disinfect the complex endodontic space [1,2]. Furthermore, the shaping phase has proven to form a smear layer haphazardly layered on the endodontic walls up to 2–5 μm in thickness [3]. Chiefly, the smear layer consists of a crystalline construction retaining remnants of pulp, dentine debris, bacteria, and their products [4,5]. The smear layer, in the event of infection, is commonly contaminated and maintains microorganisms inside the dentinal tubules, thus restricting the superior infiltration of irrigant liquids and intracanal medications [5,6]. Moreover, the smear layer renders the penetration of the obturation sealer more difficult, leading to increased coronal and apical microleakage [7]. Accordingly, eradicating the smear layer is fundamental to acquiring sufficient disinfection, not only in the main root canal but even in the lateral anatomies compromising lateral canals, loops, micro connections, isthmuses and even the dentinal tubules [6,8].

Previous research has illustrated that smear layer formation is more evident in the canal apical third. It is particularly tough to disinfect this area thoroughly as a consequence of the hidden anatomical complexity [9,10]. As specified by the input gained from a systematic review, endodontic treatment outcomes are enhanced if the smear layer is eliminated [3]. Furthermore, prior studies have investigated the implication of chemo-mechanical debridement strategies in clearing the smear layer’s attached inorganic and organic contents [11,12]. With this in mind, a standard smear layer removal protocol was proposed by applying sodium hypochlorite (NaOCl) pursued by ethylenediaminetetraacetic acid (EDTA) for 1 min [13]. Various factors determine the cleaning effectiveness and smear removal from the endodontic system.

The latest studies [4,8,14] have highlighted a novel root canal irrigation technique, 3D cleaning, which can offer a possible alternative to traditional syringe needle irrigation. The 3D cleaning technique involves the ultrasonic activation of NaOCl heated inside the root canal. Additionally, the proclaimed advantages of this novel technique are several: low cost, more pulp tissue dissolution, more antibacterial activity, and deeper irrigant penetration. Therefore, this work evaluated root canal cleanliness after performing the 3D cleaning protocol utilising scanning electron microscopy. The null hypothesis in the present work was that no statistically significant difference in debris and smear layer construction would happen when using the 3D cleaning technique compared to traditional syringe needle irrigation.

## 2. Materials and Methods

The current study was permitted by the Declaration of Helsinki guidelines and authorised by the Institutional Review Board: (University of Naples, Federico II, Italy), approval code: 55/21, approval date: 28 April 2021 (University of Naples).

Extracted lower single-rooted premolars (*n* = 30) were chosen for this research. These mandibular premolars were extracted as part of an orthodontic treatment plan and were irrelevant to the present work. Informed consent was acquired from the patients.

The exclusion criteria for selecting the teeth were fracture, crack, resorption, peri-apical lesion, previously treated tooth, history of trauma, immature apex and calcification. Inclusion criteria were closed apices, normal anatomy, single root canal, curvatures less than 10° according to the Schneider system (by buccolingual and mesiodistal radiographic analysis) [15] and sound teeth extracted for orthodontic purposes.

Immediately after extraction, the periodontal tissues on the external surface of the teeth were removed. Then, the specimens were preserved in physiological saline in separate vials at +4 °C until the experiment [5].

Before the experiment started, all the roots were randomly divided into three groups of ten specimens each.

The teeth were decoronated at the cementoenamel junction to obtain roots of standardised length (18 mm). A size 10 K-type file was inserted into each canal until it was seen through the apical foramen. The working length was established by subtracting 0.5 mm from this measurement.

The apices were then closed using wax to mimic the periodontal ligament. The canals were shaped with nickel-titanium rotary instruments (Hyflex EDM, Coltene/Whaldedent Inc., Cuyahoga Falls, OH, USA). Only the 10/0.05 and 20/0.05 instruments of Hyflex EDM were used to prepare the canals to the full working length. This step was conducted intentionally to ensure minimal preparation of the root canals.

Throughout the canal instrumentation, irrigation was performed with 3% NaOCl (Canal pro, Coltene/Whaldedent Inc., Cuyahoga Falls, OH, USA) using a side-vented 30 G needle in a syringe. A total of 5 mL of NaOCl were used for each tooth and refreshed every minute. The root canals were then rinsed with sterile saline.

At the end of the shaping phase, different protocols of irrigant activation of NaOCl were used. Teeth were assigned to groups by a random allocation sequence generated by software (Random Allocation Software 2.0) and remained concealed from the investigators.

Group (1): A measure of 3 mL of 17% EDTA (Canal pro, Coltene/Whaldedent Inc., Cuyahoga Falls, OH, USA) for 1 min was used, followed by 3 mL of sterile saline. Delivering the irrigants in the canals was conducted using a side-vented 30 G needle. Finally, traditional irrigation was performed. An endodontic needle reached 2 mm shorter than the working length, and 5 mL of NaOCl was used. A measure of 2 mL of distilled water was used as the final flush, and paper points were used to dry the canals.

Group (2): A measure of 3 mL of 17% EDTA for 1 min was used, followed by 3 mL of sterile saline. The irrigants were inserted into the canals using a side-vented 30 G needle. Finally, the 3D cleaning technique was performed:

NaOCl was delivered 2 mm shorter than the working length.

System-B Heat Source (Analytic Endodontics, Orange, CA, USA) adjusted at 180 °C was employed with an X-fine tip (30/04) 3 mm away from the working length. The tip was not in contact with the canal walls. It was activated for 8 s, followed by 20 s of ultrasonic activation using the Ultra Smart AI (Coxo, Fushan, China) size 25.00. The complete activation process was repeated five times, and NaOCl was renewed with a fresh solution per cycle. A total of 5 mL of NaOCl was utilised. Two mL of distilled water was used as the final flush, and paper points were used to dry the canals.

Group (3): saline solution (control): an endodontic needle reached 2 mm shorter than the working length, and 5 mL of saline was used. A measure of 2 mL of distilled water was used as the final flush, and paper points were used to dry the canals.

After completion of the experiments, two longitudinal troughs were drilled on each root’s lingual and buccal surfaces, utilising a diamond bur with a high-speed water-cooled handpiece to simplify vertical splitting. Following that, every specimen was immersed in liquid nitrogen instantly after root canal preparation and separated forcibly in a longitudinal way in two halves by a stainless-steel chisel. Next, all the specimens were made ready for SEM analysis; this step included allowing the samples to air-dry overnight in a desiccator at room temperature, sputter-coated with gold and prepared for SEM analysis (EVO MA 10 Carl Zeiss SMT AG, Aalen, Germany).

**Figure 1:**
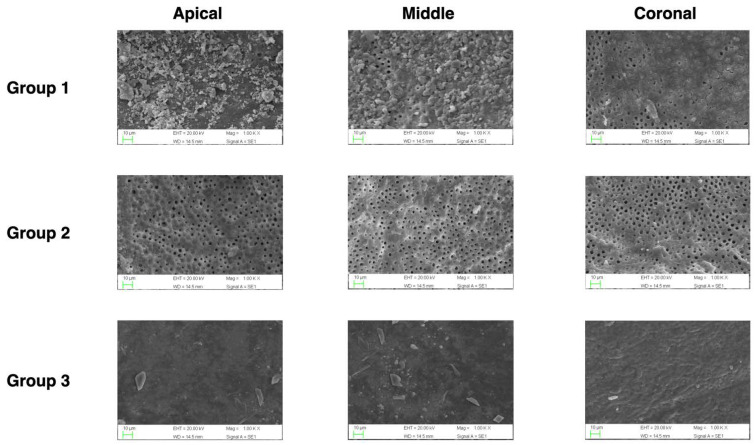
Representative samples of scanning electron microscope images of the coronal, middle and apical thirds after irrigation of the control group, traditional irrigation group and 3D Cleaning group (1000× magnification).

SEM images were acquired at a magnification of ×1000 (Figure 1), and 30 photo-micrographs were shot in three separate zones (10 for the area; coronal, middle, and apical third of the root canal).

Blindly, three trained operators scored the presence or absence of debris and smear layer on the surface of the root canal at the coronal, middle, and apical portion of each canal. The rating system used was proposed by Hulsmann et al. [16], and the criteria for scoring were reported as follows [17]:

**Scores of the debris**
  **Score 1**: Clean root canal walls with only a few small debris particles.
  **Score 2**: Few small aggregations of debris.
  **Score 3**: Many aggregations of debris covering <50% of the root canal walls.
  **Score 4**: >50% of the canal walls covered by debris.
  **Score 5**: Total or nearly entire root canal walls covered by debris.
**Scores of the smear layer**
  **Score 1**: No smear layer, and orifices of dentinal tubules are open.
  **Score 2**: Small quantity of smear layer is present, and some dentinal tubules are open.
  **Score 3**: A homogenous smear layer conceals the root canal walls, with just a few open dentinal tubules.
  **Score 4**: Entire root canal wall covered by a homogenous smear layer, no open dentinal tubules.
  **Score 5**: The heavy, homogenous smear layer covers the entire root canal walls.

## 3. Statistical Analysis

The sample size was calculated using G Power Software with an effect size of 0.4 and α = 0.05 [5]. A total of 30 samples were required, 10 per group, to set the power of 80%.

Statistical analysis was performed with a standard statistical software package (SPSS, version 28.0; SPSS IBM, Armonk, NY, USA). Debris and smear layer scores were individually registered. Explicatory statistics for ordinal data, comprising the median, 25th and 75th percentiles were calculated for each group.

Data were analyzed with a nonparametric analysis of variance (Kruskal–Wallis) among the groups tested and among the thirds of the canals. The level of significance was set at *p* < 0.05.

## 4. Results

Means, medians, percentiles, and minimum and maximum scores per group are shown in Table 1 and Table 2. The means and the medians of debris score were lower in Group 2 (3D Cleaning technique) compared with Group 1 (traditional irrigation). The Kruskal–Wallis non-parametric test show that the differences of the medians are statistically significant (*p* < 0.05). Similar results were achieved when considering the smear layer’s residual amount on the root canal’s surface with the medians of Group 1 that were higher than those in Group 2 (*p* <0.05). Thus, the null hypothesis of the study should be rejected. Comparing the three-thirds of the root canal, the test showed no statistically significant differences between the thirds in debris and smear layer groups (*p* > 0.05).

## 5. Discussion

This research aims to assess the difference in smear layer and debris removal using the 3D Cleaning technique and traditional syringe needle irrigation.

The outcome of endodontic therapy is founded on three major clinical steps: mechanical shaping, chemical cleaning and finally, the filling of the entire endodontic space [8,18,19]. Moreover, the major aim of root canal treatment is to decline or eliminate bacteria and their byproducts from the complicated endodontic area and preclude future recontamination [20,21].

Chemical disinfection is carried out using chemical irrigants, which are crucial to facilitate the disinfection and are critical for the endodontic treatment’s success [8]. Another important factor that should not be underestimated is the cytotoxic effects of the irrigants involved in the treatment [22,23].

Whereas root canal shaping cannot successfully eradicate microorganisms from the endodontic system, the contemporary Ni-Ti files cause the formation of a smear layer on the root canal walls [6]. Irrigating solutions are required to enhance the root canals’ cleaning and the removal of debris [11,14]. Presently, sodium hypochlorite (NaOCl) is the most typically used irrigant, owing to its innumerous benefits (antimicrobial activity, the capacity to dissolve pulp tissue, lubricating activity, flushing disposal of debris from the canals, economics and availability). Although NaOCl is extremely effective in antimicrobial action, it does not affect the removal of the smear layer from the dentin walls [24]. Rather, ethylenediaminetetraacetic acid (EDTA) is valued for its chelating effect on inorganic tissue and its decalcifying power [24].

A side-vented needle was used in this study to deliver the irrigants to simulate a clinically safe, realistic situation. An open-ended needle was avoided as the flow is directed towards the apical foramen, and pressure is developed with an associated risk of irrigant extrusions. Furthermore, even at low velocity, the apex is associated with a slight pressure of the irrigant, making closed-ended needles more secure in terms of evading any hazards—for example, sodium hypochlorite accidents [25]. 

Irrigant activation techniques can improve the cleanliness of the root canal walls. Currently, the most used approach for activating irrigants is ultrasonic technology, which consists of the activation of irrigants by ultrasonic cordless devices (20–42 kHz). This procedure permits an intense stirring of the irrigant through a phenomenon described as acoustic streaming. The technique mentioned above causes higher antimicrobial action and a more prominent tissue dissolution [8].

Likewise, “Laser” can be considered another promising technique in the detersion of the root canal system. However, it has some limitations linked to increased equipment expense and the irrigant apical extrusion hazard [26].

As aforementioned, the efficacy of irrigants can be enhanced by employing activated irrigation strategies. One simple method of activating NaOCl is pre-heating it to a temperature of 50 °C. Moreover, heated NaOCl has been shown to have superior antimicrobial and tissue-dissolving properties [4,8,15]. However, delivering pre-heated NaOCl has its disadvantages. The body can rapidly buffer the NaOCl, thus mitigating its advantages [8].

Regarding the “Intra-canal regulated heating irrigant activation”, heated NaOCl improves its antimicrobial, dissolution and lubrication effects. However, in order to achieve this objective, the NaOCl must be heated straight within the root canal by a temperature-regulated tip [14,27]. A study by Iandolo A. et al. [4] showed how the internal heating technique, even without using EDTA, achieved good results for smear layer and debris removal.

The presented research evaluated combining two well-studied techniques: the ultrasonic activation of heated NaOCl [15]. This novel technique has many advantages, and these research results also showed a promising new action: smear layer and debris removal.

Several in vitro studies in the literature have used SEM analysis. SEM is an outstanding device for this examination. However, it has numerous drawbacks. For instance, specimens must be prepared in sections for examination, and it is a subjective qualitative evaluation that can be quantified by multiple scoring systems [28]. Hence, SEM analysis alone does not permit a proper longitudinal observation of the dentin morphology; evaluation with Micro-CT analysis is crucial. Furthermore, the latter is more reliable and offers an essential requirement for sampling and analysing the smear layer removal methods [29]. Another limitation of this research was using single-rooted teeth with curvatures less than 10° for evaluation. Therefore, further studies should address assessing teeth with complicated root canal anatomical configurations.

## 6. Conclusions

The 3D cleaning technique, based on the ultrasonic activation of intracanal-heated NaOCl, shows to be more effective than the traditional irrigation method in obtaining clean canal walls. Future research, such as CLSM (Confocal Laser Scanning Microscopy), Micro-CT analysis and histological study, should be employed to confirm this study’s conclusion.

## Figures and Tables

**Table 1:** Means, medians, 25 and 75 percentiles per group, D: Debris.

Group		D Coronal	D Middle	D Apical	D Total
1	Mean	3.55	4.45	4.55	4.18
	N	11	11	11	33
	Median	4	4	5	4
	25 Percentile	3	4	4	4
	75 Percentile	4	5	5	5
2	Mean	1	1.18	1.36	1.18
	N	11	11	11	33
	5	1	1	1	1
	4	1	1	1	1
	5	1	1	1	1
3	Mean	5	5	5	5
	N	11	11	11	33
	Median	5	5	5	5
	25 Percentile	5	5	5	5
	75 Percentile	5	5	5	5
Total	Mean	3.18	3.55	3.64	
	N	33	33	33	
	Median	4	4	5	
	25 Percentile	1	1	2	
	75 Percentile	5	5	5	

**Table 2:** Means, medians, 25 and 75 percentiles per group, SL: Smear Layer.

Group		SL Coronal	SL Middle	SL Apical	SL Total
1	Mean	3.73	4.18	4.55	4.15
	N	11	11	11	33
	Median	4	4	5	4
	25 Percentile	3	4	4	4
	75 Percentile	4	5	5	5
2	Mean	1	1.18	1.27	1.15
	N	11	11	11	33
	Median	1	1	1	1
	25 Percentile	1	1	1	1
	75 Percentile	1	1	2	1
3	Mean	5	5	5	5
	N	11	11	11	33
	Median	5	5	5	5
	25 Percentile	5	5	5	5
	75 Percentile	5	5	5	5
Total	Mean	3.24	3.45	3.61	
	N	33	33	33	
	Median	4	4	5	
	25 Percentile	1	1	1.5	
	75 Percentile	5	5	5	

## References

[1] Tosco V., Monterubbianesi R., Aranguren J., Memè L., Putignano A., Orsini G. (2023). Evaluation of the Efficacy of Different Irrigation Systems on the Removal of Root Canal Smear Layer: A Scanning Electron Microscopic Study. Appl. Sci..

[2] Teja K.V., Ramesh S., Battineni G., Vasundhara K.A., Jose J., Janani K. (2022). The effect of various in-vitro and ex-vivo parameters on irrigant flow and apical pressure using manual syringe needle irrigation: Systematic review. Saudi Dent. J..

[3] Torabinejad M., Handysides R., Khademi A.A., Bakland L.K. (2002). Clinical implications of the smear layer in endodontics: A review. Oral Surg. Oral Med. Oral Pathol. Oral Radiol. Endod..

[4] Violich D.R., Chandler N.P. (2010). The smear layer in endodontics—A review. Int. Endod. J..

[5] Rajamanickam K., Teja K.V., Ramesh S., AbuMelha A.S., Alkahtany M.F., Almadi K.H., Bahammam S.A., Janani K., Choudhari S., Jose J. (2022). Comparative Study Assessing the Canal Cleanliness Using Automated Device and Conventional Syringe Needle for Root Canal Irrigation-An Ex-Vivo Study. Materials.

[6] Martina S., Pisano M., Amato A., Abdellatif D., Iandolo A. (2021). Modern rotary files in minimally invasive endodontics: A case report. Front. Biosci. Elite.

[7] Çobankara F.K., Adanır N., Belli S. (2004). Evaluation of the influence of smear layer on the apical and coronal sealing ability of two sealers. J. Endod..

[8] Iandolo A., Abdellatif D., Amato M., Pantaleo G., Blasi A., Franco V., Neelakantan P. (2020). Dentinal tubule penetration and root canal cleanliness following ultrasonic activation of intracanal-heated sodium hypochlorite. Aust. Endod. J..

[9] Iandolo A., Dagna A., Poggio C., Capar I., Amato A., Abdellatif D. (2019). Evaluation of the actual chlorine concentration and the required time for pulp dissolution using different sodium hypochlorite irrigating solutions. J. Conserv. Dent..

[10] Park E., Shen Y.A., Haapasalo M. (2012). Irrigation of the apical root canal. Endod. Top..

[11] Alamoudi R.A. (2019). The smear layer in endodontic: To keep or remove—An updated overview. Saudi Endod. J..

[12] Di Spirito F., Pisano M., Caggiano M., Bhasin P., Lo Giudice R., Abdellatif D. (2022). Root Canal Cleaning after Different Irrigation Techniques: An Ex Vivo Analysis. Medicina.

[13] Kharouf N., Pedullà E., La Rosa G.R.M., Bukiet F., Sauro S., Haikel Y., Mancino D. (2020). In Vitro Evaluation of Different Irrigation Protocols on Intracanal Smear Layer Removal in Teeth with or without Pre-Endodontic Proximal Wall Restoration. J. Clin. Med..

[14] Iandolo A., Abdellatif D., Pantaleo G., Sammartino P., Amato A. (2020). Conservative shaping combined with three-dimensional cleaning can be a powerful tool: Case series. J. Conserv. Dent..

[15] Schneider S.W. (1971). A Comparison of Canal Preparations in Straight and Curved Root Canals. Oral Surg. Oral Med. Oral Pathol..

[16] Metzger Z., Teperovich E., Cohen R., Zary R., Paqué F., Hülsmann M. (2010). The self-adjusting file (SAF). Part 3: Removal of debris and smear layer—A scanning electron microscope study. J. Endod..

[17] Rödig T., Hülsmann M., Kahlmeier C. (2007). Comparison of root canal preparation with two rotary NiTi instruments: ProFile. 04 and GT Rotary. Int. Endod. J..

[18] Iandolo A., Pantaleo G., Malvano M., Simeone M., Amato M. (2016). Nonsurgical management of complex endodontic cases with several periapical lesions: A case series. G. Ital. Endod..

[19] Iandolo A., Simeone M., Riccitiello F. (2012). The preparation of coronal isthmus is a fundamental step for long term success. G. Ital. Endod..

[20] Crozeta B.M., de Souza L.C., Silva-Sousa Y.T.C., Sousa-Neto M.D., Jaramillo D.E., Silva R.M. (2020). Evaluation of Passive Ultrasonic Irrigation and GentleWave System as Adjuvants in Endodontic Retreatment. J. Endod..

[21] Iandolo A., Amato A., Martina S., Abdellatif D.A., Pantaleo G. (2020). Management of severe curvatures in root canal treatment with the new generation of rotating files using a safe and predictable protocol. Open Dent. J..

[22] Özdemir O., Kopac T. (2022). Cytotoxicity and biocompatibility of root canal sealers: A review on recent studies. J. Appl. Biomater. Funct. Mater..

[23] Özdemir O., Kopac T. (2022). Recent Progress on the Applications of Nanomaterials and Nano-Characterization Techniques in Endodontics: A Review. Materials.

[24] Plotino G., Cortese T., Grande N.M., Leonardi D.P., Di Giorgio G., Testarelli L., Gambarini G. (2016). New Technologies to Improve Root Canal Disinfection. Braz. Dent. J..

[25] Boutsioukis C., Verhaagen B., Versluis M., Kastrinakis E., Wesselink P.R., van der Sluis L.W. (2010). Evaluation of irrigant flow in the root canal using different needle types by an unsteady computational fluid dynamics model. J. Endod..

[26] Dioguardi M., Gioia G.D., Illuzzi G., Laneve E., Cocco A., Troiano G. (2018). Endodontic irrigants: Different methods to improve efficacy and related problems. Eur. J. Dent..

[27] Amato M., Pantaleo G., Abtellatif D., Blasi A., Gagliani M., Iandolo A. (2018). An in vitro evaluation of the degree of pulp tissue dissolution through different root canal irrigation protocols. J. Conserv. Dent..

[28] Hülsmann M., Rümmelin C., Schäfers F. (1997). Root Canal Cleanliness after Preparation with Different Endodontic Handpieces and Hand Instruments: A Comparative SEM Investigation. J. Endod..

[29] Freire L.G., Iglecias E.F., Cunha R.S., dos Santos M., Gavini G. (2015). Micro–Computed Tomographic Evaluation of Hard Tissue Debris Removal after Different Irrigation Methods and Its Influence on the Filling of Curved Canals. J. Endod..

